# Aspects Regarding the Innovative Conceptual Design for Children’s Recreational Areas

**DOI:** 10.3390/children9081240

**Published:** 2022-08-17

**Authors:** Bogdan Bucur, Andreea Ban, Sorin Vlase, Arina Modrea

**Affiliations:** 1 Department of Engineering and Technology of Information, George Emil Palade University of Medicine, Pharmacy, Science and Technology of Targu Mures, Str. Gh. Marinescu 38, 540142 Târgu Mureș, Romania; 2 Department of Mechanical Engineering, Transilvania University of Brasov, B-dul Eroilor, 29, 500036 Brasov, Romania

**Keywords:** concept design, innovation, creativity, systemic analysis, parameterized design

## Abstract

Conceptual design approaches the definition of an innovative concept design applied to a product intended for children’-=s recreational area, corresponding to a personal profile located around the age of childhood, namely aged 2 to 5. The case study presented highlights the development of an innovative concept for children’s recreational areas, to be precise the design, and the conceptual design applied to a roto-pendular carousel for children’s recreational areas. The working method aims at identifying the chronological stages of development and the conceptual design, starting from the idea, prospectographic research, analysis of constructive solutions, and finally the materialization of the assisted design in detail, applied to the new concept. Based on the prospectographic study, several constructive variants are highlighted, at the draft level, and after the analysis, the optimized variant of the carousel concept is established that should match the functions and characteristics pursued and imposed on the new concept. Following the justification of the choice of the optimal variant of the concept, the model is designed in an assisted environment in order to follow aspects related to technology associated with each component of the whole assembly, as well as aspects related to ergonomics, safety in exploration and proportion associated to the typology and to the age criteria of children.

## 1. Introduction

The industry responsible for the creation of products for children’s recreational areas is constantly growing, due to the development and continuous appearance of as many such areas as possible, both at the level of recreational parks and at the development of closed spaces for parties and children’s socialization in different age groups. Conceptual design is an innovative concept design applied to a product intended for children [1,2,3,4,5,6,7,8].

When we refer to children’s education, the most effective way for the development of communication abilities and logical thinking can be achieved through games, [9,10]. Their effectiveness lies in the fact that all children take part in their development, they make efforts to think, to express themselves, without realizing this, considering that they are playing.

As an activity, games contribute to the development of a positive attitude towards work and learning. It is an effective method that contributes to the development of language, an activity through which we achieve communication and develop our thinking. Games impose a state of relaxation, and good mood, helping children to form their ability to create. Through such activities, they can learn how to express themselves, using a language as correct, beautiful, and nuanced. In the day-to-day activity of the child, the game obviously plays an important role. When children play, they satisfy their need for activity, for acting with real or imaginary objects, and for translating into different roles and situations that bring them closer to the surrounding realities. Additionally, the prototype of the roto-pendular carousel represents one of the best ways that can help children overcome their shyness when interacting with others.

Additionally, the game is a faithful mirror of the children’s personalities as a whole. Thus, it is known that when playing, the child puts into action the possibilities arising from his/her particular structure, transforms into facts the virtual potencies that appear successively on the surface of his/her being, assimilates, develops, combines, and complicates them, thus coordinating his/her becoming. Therefore, the game develops the latent functions of the psyche [9].

Another competence that the game can develop very nicely is interpersonal relationship competence and civic competence. Fortunately, a lot of games can be created so that this skill can be developed. In “Improving the Social Communication Skills of At-Risk Preschool Children in a Play Context” Lesley A. Craig-Unkefer and Ann Kaiser emphasize the importance of games and social interaction: “Although the relationship between language and social skills is complex, interventions that increase both skills may increase the likelihood of children’s competent participation in play-based social interactions” [10,11].

In general, most playgrounds have approximately the same play equipment: swings, slides, sandpits, and climbing frames. In general, children are attracted to the novelty elements present in the playground, and especially they are attracted to figures associated with characters from the world of cartoons that are very well stylized and integrated into the architecture of the space, so, creating at the same time an atmosphere of good mood for socializing and entertainment.

Thus, the world of cartoon characters presents a source of inspiration for the design and adaptation of this equipment intended for playgrounds, while respecting the safety rules of children during play.

Taking these aspects into account, the paper proposes a staged development regarding the design of the prototype concept of a carousel, which will be analyzing synoptic, ergonomic, aesthetic functional, and safety aspects.

## 2. General Aspects

According to Wikipedia [12,13,14], the carousel (from French: carousel) is a fun, purpose installation composed of a circular platform rotating around a vertical shaft, on the periphery of which animal figurines or vehicles are mounted, provided with places for the user audience. Originally, the carousel used wooden horse figurines, in a fixed position or sliding along a vertical rod, which imitated during operation the feeling of a gallop, hence the traditional name of “călușei”, used in Romanian (other names: “căișori”, “ringhispil”).

The first carousel was created in 500 B.C. in Rome, its main purpose was to bring joy related to play, both for children and adults. This carousel consisted of baskets in which children or adults were seated, suspended from a central pole.

**Figure 1:**
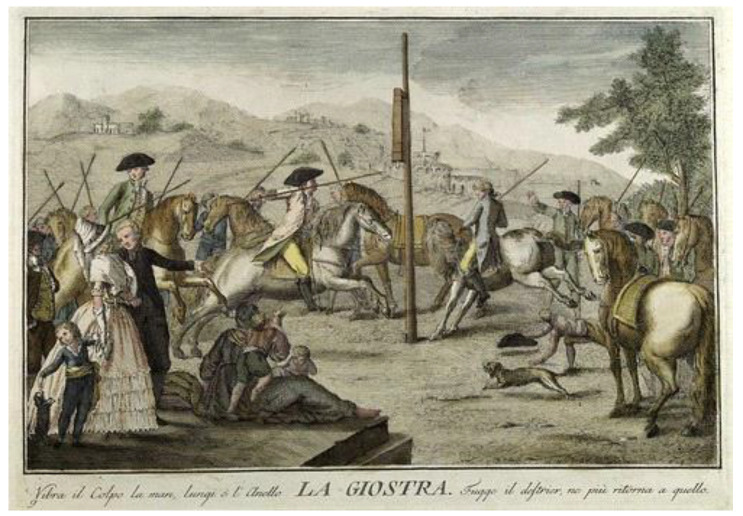
Semi-carousel of the seventeenth century (France) [12].

**Figure 2:**
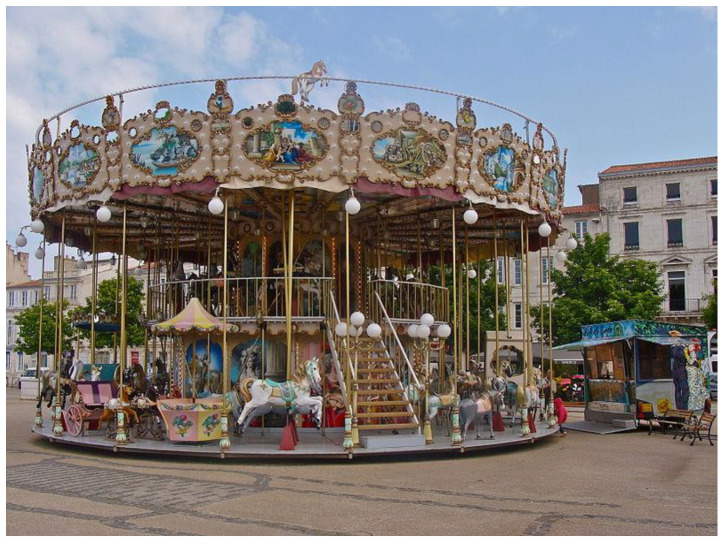
Carousel of the nineteenth century (France) [13].

In the year 1780, the oldest stationary carousel in the world was built, and its construction continued until 1896. In the year 1876, the first car was built on a platform that operated in an amusement park for children (Figure 1 and Figure 2). Between 1890 and 1920, the first carousel operated by a classic steam engine was built and is preserved today as a very rare specimen.

Nowadays, carousels have various shapes and are mostly intended for children’s recreational areas and playgrounds, developing at the same time a variety of sizes with multiple functions and complex characteristics, from the simplest systems intended for young children (aged 2–7) to adults developing dynamic forces up to 4G (extremely dangerous in terms of thrills) [13,14].

## 3. Materials and Methods

The case study proposes an analysis of the development and design of a carousel concept for the age group between 2 to 5 years old, for which the degree of danger is very low, given that the movement is made by the inertia of the body of two to four children of close age, thus achieving a couple of moments of forces to achieve the dynamic movement of the carousel concept [1,5,15].

**Figure 3:**
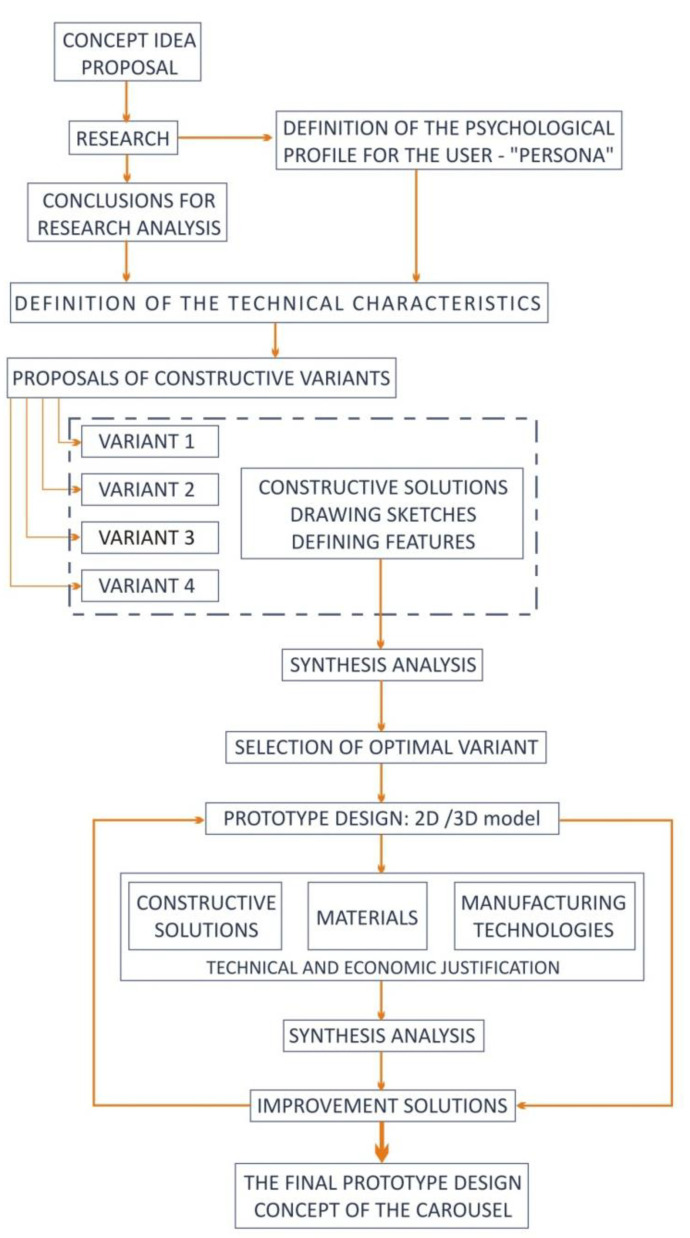
The methodology for analyzing and defining ideas.

In Figure 3, you can see the methodology for analyzing and defining ideas regarding the approach and prototype design of the carousel concept.

Prospectographic documentation includes new models, and we focus on popular carousel models, namely those provided with side seats, which we often find in the amusement park. They are provided with safety belts that allow obtaining a fixed position during operation. Additionally, the attention paid to these details differentiates the studied concepts. The visual aspect is the first element that attracts the children’s attention, and for this reason, the products are highlighted by a vast chromatic palette, which highlights strong shades of red, blue, green, yellow, or purple [9].

The color combinations attributed to the play equipment, develop visual attention and exert a psychological impact on children, creating at the same time an atmosphere of positive energy and enthusiasm [10,11]. It should be noted several defining characteristics associated with colors, which are recommended as follows:

The *red color* is the best perceived color for an apathetic, slow, or modest child, and it will contribute to increasing communication skills as well as improving mood;

The *yellow color* conveys sensory, joy, and happiness for children, stimulates the mind to concentrate, inspire, and obey;

The *green color* develops children’s creativity and character, arouses interest in the world around them, raises self-esteem and self-confidence, and inspires courage and positive impact, especially for shy and passive children;

The blue color conveys the feeling of calmness, it will cancel anxiety and depression, respectively, eliminate and neutralize the tension of excitement;

The *purple color* is a calming shade, it will convey balance, positive energy, and enthusiasm, attracting harmony and health.

Obviously, the colors have a great impact on children’s psyche, so choosing the right color shades applied to the concept, subliminally, determines the emotional and affective states of children which can be kept under control to calm or inspire.

The sizes and shapes are also diversified, precisely to provide versatile, customizable constructive variants that gradually make up a favorable environment in which children can play safely [16]

Table 1 identifies the main equipment that defines the prospectographic research study. Since the beginning of play areas specially designed for children, the carousel has been a constant companion of children’s play. From the simplest shapes and moving up to the new trends in the design and realization of carousels, they occupy an important niche in play spaces. A carousel gives children pleasure, comfort, and relaxation.

Following the prospectographic study carried out, some conclusions can be drawn regarding the outline of the structure of the design concept proposed for definition. It highlights color characteristics, sizing, number of users associated with the concept, as well as materials structure and color palette applied to the competitive product.

Thus, based on the prospectographic analysis carried out on a series of studied design concepts, a specification can be described that includes the following aspects that will be identified for the design concept applied to the proposed prototype [1,16]. These aspects will be presented in 3 or 4 suggested variants from which the optimized variant will be chosen while justifying the chosen constructive variant.

The location of the carousel is carried out in environmental parks as well as in closed play spaces, intended for socialization and parties for children of pre-school age, being also a novelty element associated with the equipment that is used for the children’s play spaces, combining at the same time two movements and one of rotation (centrifugal), and one pendulum (undefined directionally) [30].

## 4. Research Conclusions

Following the prospectographic research analysis, certain defining requirements of the new product to be designed and executed of the carousel prototype can be outlined in a later stage. The characteristics of shape, size, color, performance and safety in use are highlighted, as well as the materials proposed for the realization of the prototype.

Thus, the specification is outlined, which includes the necessary aspects that must be taken into account for the realization of at least four variants, proposed in accordance with the intended purpose, from which, following the synthesis analysis, the optimal prototype variant will be chosen.

## 5. Proposal Specification

- Easy placement in open spaces and playgrounds, as well as in closed play spaces, intended for socialization and parties for children;
- The combination of at least two different functions is also a novelty element associated with the equipment that is used for the children’s play spaces, combining at the same time two movements, one of rotation (centrifugal) and one pendulum (undefined directionally);
- Intended for preschool children: aged 2 to 5 years;
- The equipment is easy to place both indoors in recreational areas and outdoors in playgrounds;
- Combined materials: metal/plastics or combined with glass fiber/acrylic;
- Complementary color combinations, specific to childhood period;
- Simultaneous users: 3 children;
- The carousel can be mounted on surfaces of grass, slag, sand, concrete/asphalt, synthetic suprafete;
- Adult supervision is recommended;
- It is also easy to clean and sanitize with specific cleaning means, being resistant to abrasive chemical factors and especially to outdoor climatic factors;
- Safe space: ø 2450;
- Colors in warm, complementary tones;
- Proposal for constructive execution: variant 1, intended for 2–3 children, variant ii for only one child;
- Robust, provides a weight of up to 50 kg;
- Rigid fastening in reinforced concrete block at the lower part, and at the upper part of the helical spring it is fixed by means of a metal plate, integral with the seat of the equipment;
- Constructive variant i, for 2–3 children, maximum sizes: Ø 1200 × 1600 mm;
- Constructive variant ii, for only one child, sizes: Ø 800 × 1200 mm.
- On the basis of the proposed specification, all the stages will be established for the design and development of the new innovative product concept.

## 6. Proposed Conceptual Variants

This section may be divided into subheadings. It should provide a concise and precise description of the experimental results, their interpretation, as well as the experimental conclusions that can be drawn.

For the concept project, it is proposed to define a number of three or four constructive variants, sketched and applied to the concept, partially or completely respecting or combining the technical solutions from one variant to another, and after the analysis of the concepts to decide by justification the choice of the optimal variant of concept design prototype of the final variant of the innovative concept. For the systemic concept analysis, four constructive variants with different functions and parts are proposed, following a common line of the theme development [1,7,15,30].

The first problem that is identified is related to the thematic idea from which the product development is based, so in order to respond to this problem, sources of inspiration are identified that compete to materialize the variants. It is important to establish a common theme of all the outlined concepts in order to develop and diversify the range of proposed variants.

The idea from which one starts when defining a new concept design is sometimes more difficult to materialize in the final prototype to be realized, and for the present case study, considering who the new concept is addressed, the most indicated source of inspiration is found in the world of cartoons [31,32,33,34,35,36,37,38].

The inspiration and curiosity of finalizing the approached theme is based on the fascinating world of cartoons, characters that marked and delighted our childhood, funnily drawn in expressive colors applied to an aesthetic geometric structure and proportionally created, and also an unsecured source of inspiration dedicated and recommended for such a field: design concepts for children’s playgrounds.

Following the study and analysis of the characters in the cartoon world, it is found that a common clothing element could be identified as a source of inspiration, so the clothing element is proposed as an object that accessorizes the animated character, and is simple and common, respectively, a clothing element that covers the head: a hat, cap, beret. This clothing accessory is among the most common items of clothing identified and attributed to the characters of the cartoon world. The first aspect that should be emphasized regarding the item of clothing concerned is that when it is associated with the character, it subliminally transmits a faster identification of the character preferred by children.

**Figure 4:**
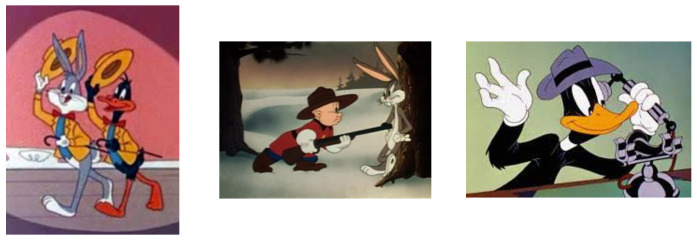
Animated characters associated with the inspiration of ideas in Variant 1 [31].

**Figure 5:**
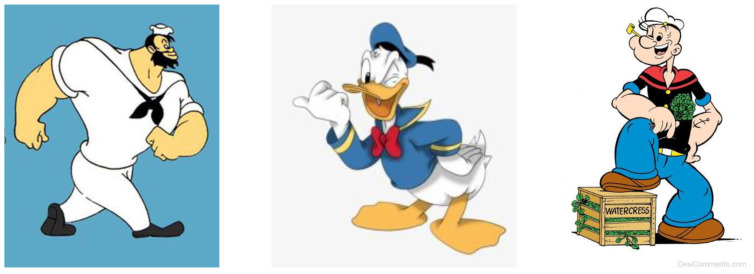
Animated characters associated with the inspiration of ideas in Variant 2 [32,33,34].

**Figure 6:**
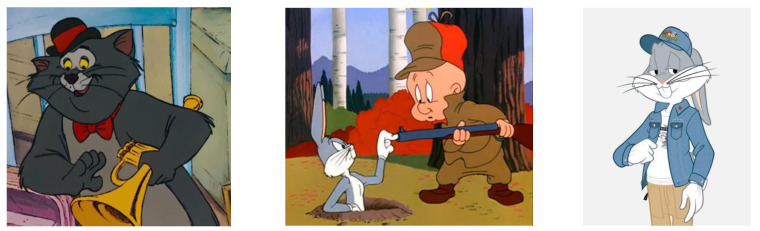
Animated characters associated with the inspiration of ideas in Variant 3 [35,36].

**Figure 7:**
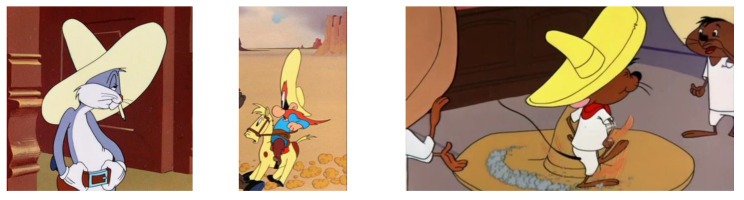
Animated characters associated with the inspiration of ideas in Variant 2 [37,38].

In Figure 4, Figure 5, Figure 6 and Figure 7, some characters belonging to the cartoon world are presented, which were the basis for the analysis of the definition of the general concept idea in the approach to the common theme of the variants proposed for analysis, in order to justify the choice of the optimal variant of the design concept, applied to the finished product, namely the carousel.

To begin with, a common theme is defined for all the variants proposed for analysis, respectively, following an analysis study on animated characters, the general theme resulted in the approach of an innovative design concept developed synoptically according to a field of clothing, hat, cap, beret, etc. Starting from these clothing accessories, the design concepts are defined, respectively, we have chosen: deep-brimmed hat, jockey cap, sailor beret, sun hat sombrero.

Following the systemic analysis, the optimal variant will be defined while establishing the technological manufacturing solutions, as well as identifying the materials applied to the components of the conceptually designed assembly.

According to the materialized ideas, the closest geometry of the proposed clothing object is identified starting from a real model, for each proposed variant for analysis and validation of the optimal variant.

## 7. Proposed Concept Sketches

### 7.1. Variant 1 / Concept Product Design

Variant 1 proposes as a synoptic model the definition of the design concept starting from a hat with a deep “brim”.

**Figure 8:**
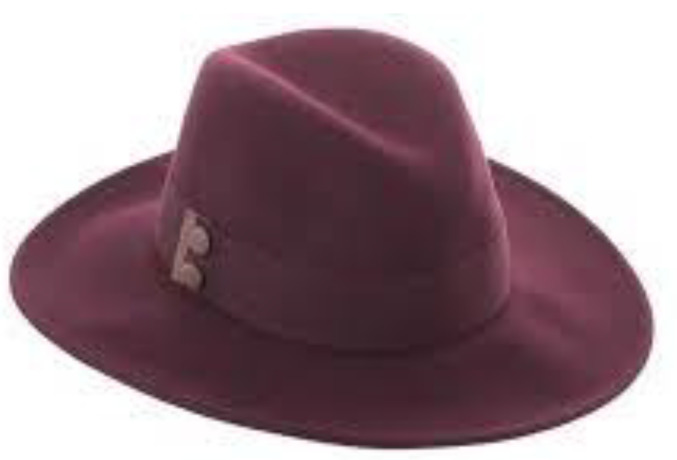
Inspirational model chosen for Variant 1 [39].

**Figure 9:**
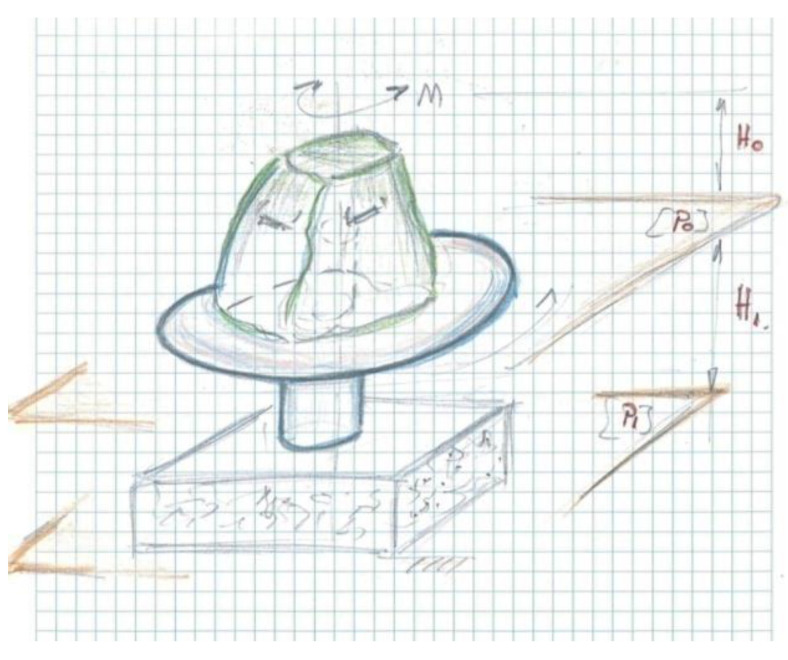
Concept sketch-design hat with deep “brim”.

In Figure 8 and Figure 9, you can see the example of the hat that was the basic idea of the conceptual inspiration for Variant 1, the design proposal for the concept product.

*The sketched pattern* presents a synoptic variant of the concept, starting from the geometry of a hat from the 1960s, having a deep “brim”, used as a support for the children’s feet, and the child’s position is vertical, on the “pointy” side of the hat there are four handles arranged in a diametrically opposed order. For this variant of the concept, it a maximum of four users are recommended, preferably an even number arranged diametrically opposed in order to balance the moments of inertia.

*The concept* can rotate around the symmetry axis on a spindle placed in the middle of the geometric axis of the hat, and the central pillar is fixed in a cement block, which is subsequently buried to the level of the “hat brim”. See plan [P_0_], above ground height (H_0_), defines an anthropometric average size of the average height of children aged 2 to 7, establishing the position of fixing the handles on the “the tip of the hat”, see Figure 9.

*Gauge dimensions:* H_0_ = 120 cm, hat diameter: d = Ф120 cm and the burial depth of the cement block is H_1_ = 50 cm. The circular support has at the top two bearings that are fixed inside the hat, one bearing is radial and the second is axial, thus allowing the recovery of forces and moments of inertia in the radial and axial direction.

*The material* proposed for the hat support is polyethylene, and the geometric shape of the hat is defined by the technology of rotoforming, resulting in an approximate geometric shape of a plate. The assembly solution on the support pillar by inserting a bushing into the rotoforming mold allows defining a non-removable assembly made up of two different materials: metal and plastic. Then, in the elongated bushing, it is allowed to assemble the rolling bearings (radial bearing, axial bearing) with the support pillar fixed in a cement block.

The proposed model performs only one function of those proposed in the specifications, namely the rotation around the geometric axis is ensured.

*Proposed colors* for the concept: purple hat structure, contrasting handles of “electric green” shade, on the support of the hat (“deep brim”), a rubberized self-adhesive tape is recommended, to prevent possible slipping of children during manual rotation of the carousel.

### 7.2. Variant 2 / Concept-Product Design

**Figure 10:**
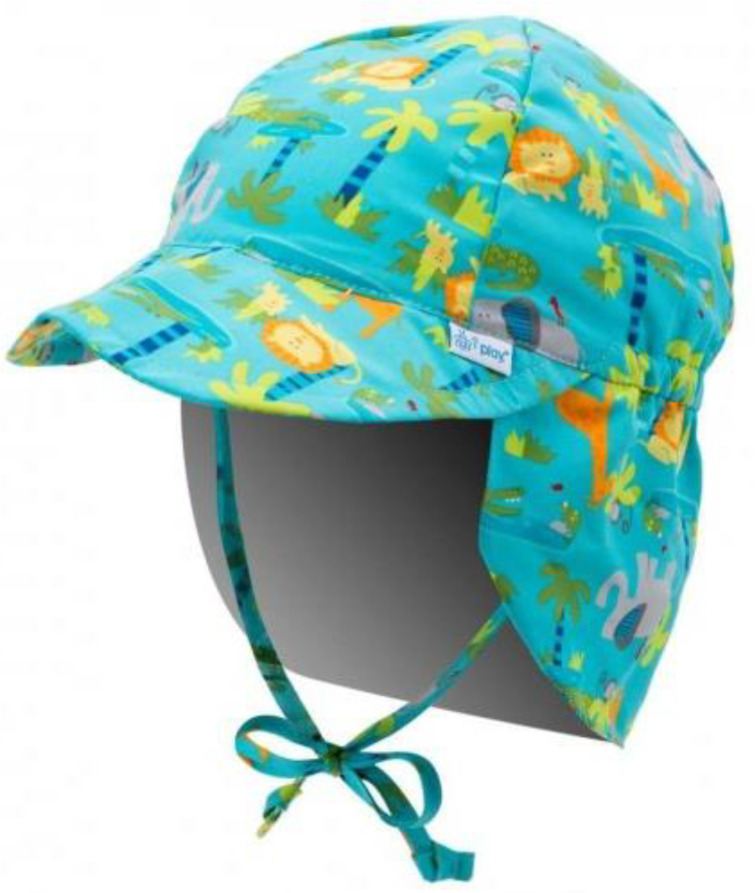
Inspirational model chosen for Variant 2 [40].

Variant 2 proposes a synoptic model as the definition of the design concept starting from the jockey cap. In Figure 10, one can identify the cap model that is the inspiration point in the development of the design concept of the carousel.

**Figure 11:**
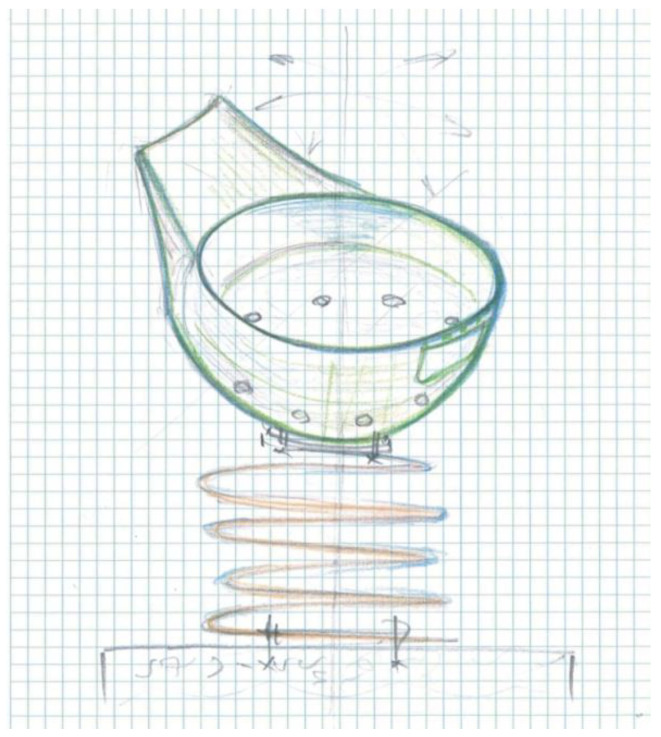
Concept sketch, jockey cap.

*The concept* allows performing pendulum movements in undefined directions linearly by the pendulum due to the elasticity of the helical spring secured to the lower part by a cement block, and the upper part is fixed to the semicircular part of the outer cavity of the jockey cap, oriented inversely (upside down). In Figure 11, the sketch proposed for the design concept for Variant 2 is highlighted.

The sketched model presents a synoptic variant of the concept, starting from the stylized configuration of a jockey cap, the idea associates a concept of a carousel for a single user.

The concept presents a higher degree of security than Variant 2, since the child is placed inside the cavity of the jockey cap and by pendulating the subassembly the “brawnian” movement occurs.

The design concept presented is limited in terms of gauge and users, with a maximum of one child, as two children can unbalance it and accidents can occur. Safety is ensured only if the child is inside the cavity of the jockey’s cap, given the fact that by the pendulum movement the trajectory cannot be identified due to the elasticity of the strained spring.

*The material* proposed for the definition of the concept is polyethylene, the model is made by a thermoforming technology, and it is fixed at the bottom by a curved metal plate according to the geometry of the concavity of the geometry of the clothing object.

### 7.3. Variant 3 / Concept-Product Design

Variant 3 proposes as a synoptic model the definition of the design concept starting from a sailor’s beret, leading us at the same time to the theme of the concept.

**Figure 12:**
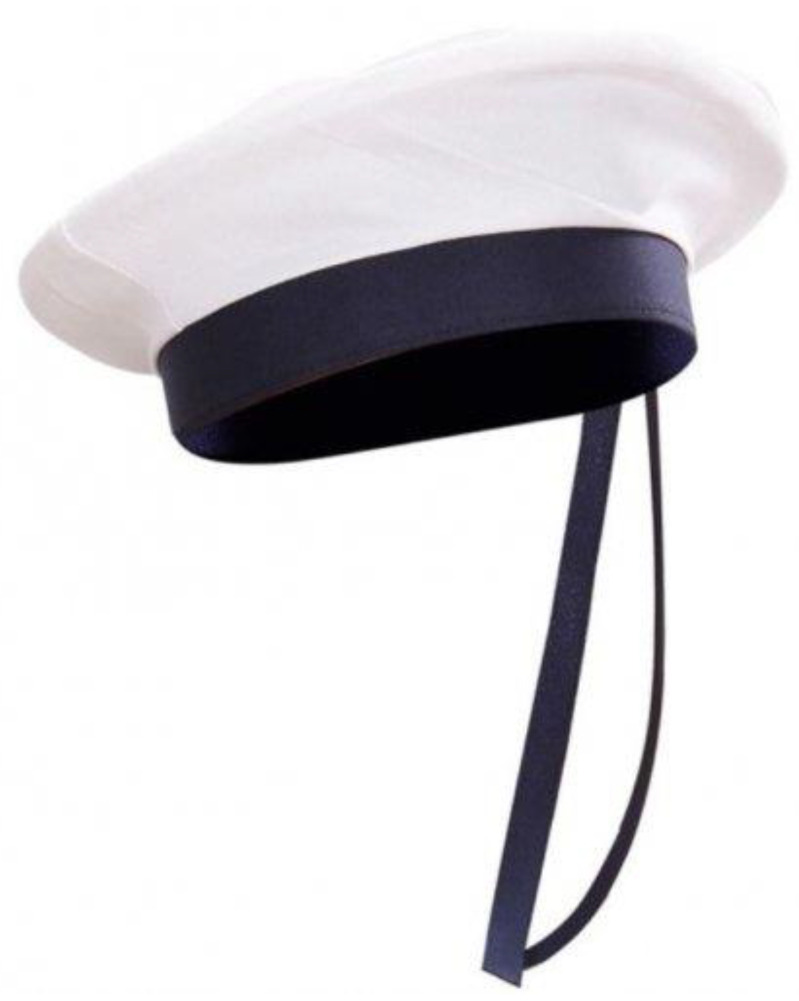
Inspirational model chosen for Variant 3 [41].

In Figure 12, one can identify the sailor beret model that is the inspiration point in the development of the design concept of the carousel.

The proposed and sketched model presents a synoptic variant of the concept, starting from the stylized configuration of a sailor’s beret and the base is the element of support for the user, around which the whole assembly rotates. The central axis is solidly tied to the base in the form of a sailor’s beret and solidly tied to the supporting platter on which a helicoidal arch is fixed, and the outside wall it is fixed to a cement block. This ensures a rotational motion and a pendular compound motion due to the elasticity of the helical spring.

**Figure 13:**
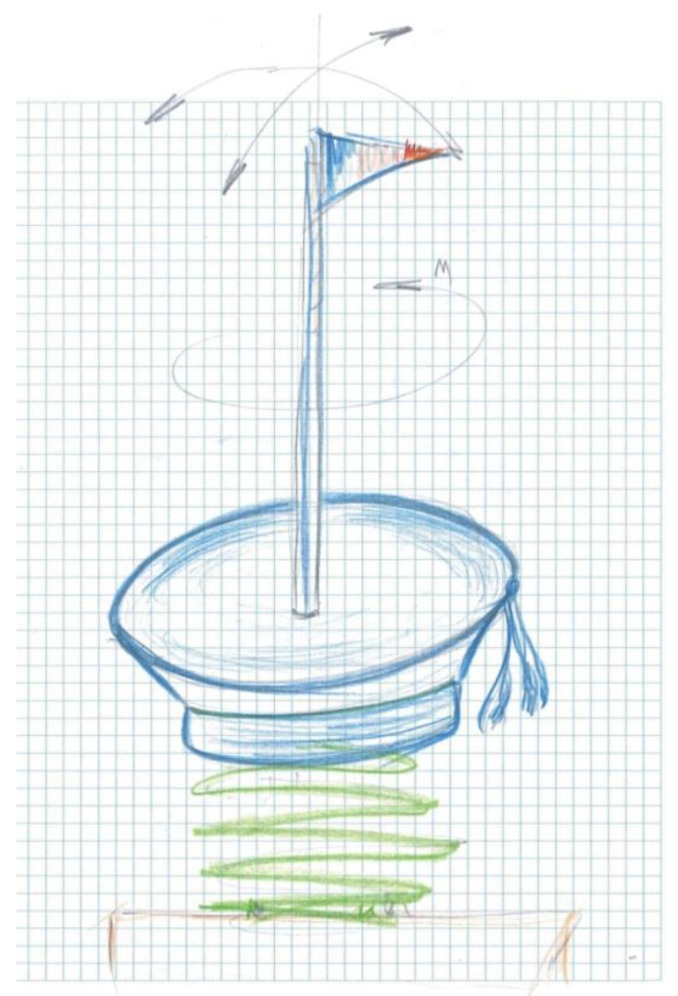
Concept sketch, design Variant 3 sailor’s beret [27].

In Figure 13 the concept is outlined, identifying particular elements.

*The concept* is limited in terms of child safety since there is a very small area of support and fixation of children on the platform of the carousel, and the size of the mast is slightly oversized to be able to fix the base on the support by means of a bearing at the lower part of the base.

*The model* is intended to be used by three to four preschool children, the sizing of the spring must be slightly rigid so as to allow the fixation of a central axis of medium height up to 1.5 m, around which the rotational movement of the carousel will be produced.

*The material* proposed for the manufacture of the concept is the fiber, and the initial geometry is defined in the form of a welded wire structure ensuring a high strength concentrated on the middle part where the central axis fixing system is identified, as well as the assembly with rolling bearings to ensure rotational movement. The central axis is fixed solidly on a board that is fixed on top of the arch with a bracket of clamp eclipses, and the arch at the bottom is fixed in a cement block, which is buried at ground level.

### 7.4. Variant 4 / Product Design Concept

Variant 4 suggests a sun hat, the “sombrero” type, as a synoptic model to define the design concept. It is a model liked by children especially as it illustrates a character present in many cartoons that brought us so much joy in our childhood.

**Figure 14:**
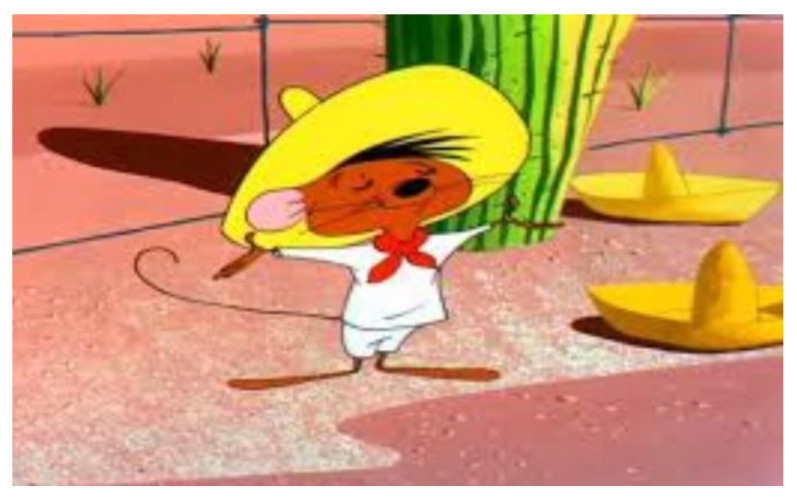
Cartoon character Speedy Gonzales [42].

In Figure 14, the image illustrates the character that inspired the development of the concept, and the sombrero sun illustrates a novel idea, which constitutes the originality in the initiation of the design concept associated with the proposed carousel. 

In Figure 14 you can see the image of the cartoon character that inspired the development of the concept (“Speedy Gonzales”, classical cartoon’s personage), the sombrero hat illustrates a novel idea, which constitutes the originality in the initiation of the design concept associated with the prototype of the carousel. 

**Figure 15:**
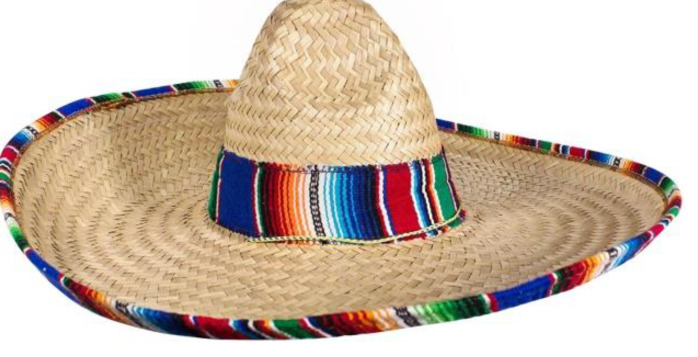
Inspirational model chosen for Variant 4, the sombrero hat [43].

In Figure 15, you can see the sun hat, the sombrero type; we can notice the details that constitute the model of the design concept proposed for Variant 4 of the carousel.

**Figure 16:**
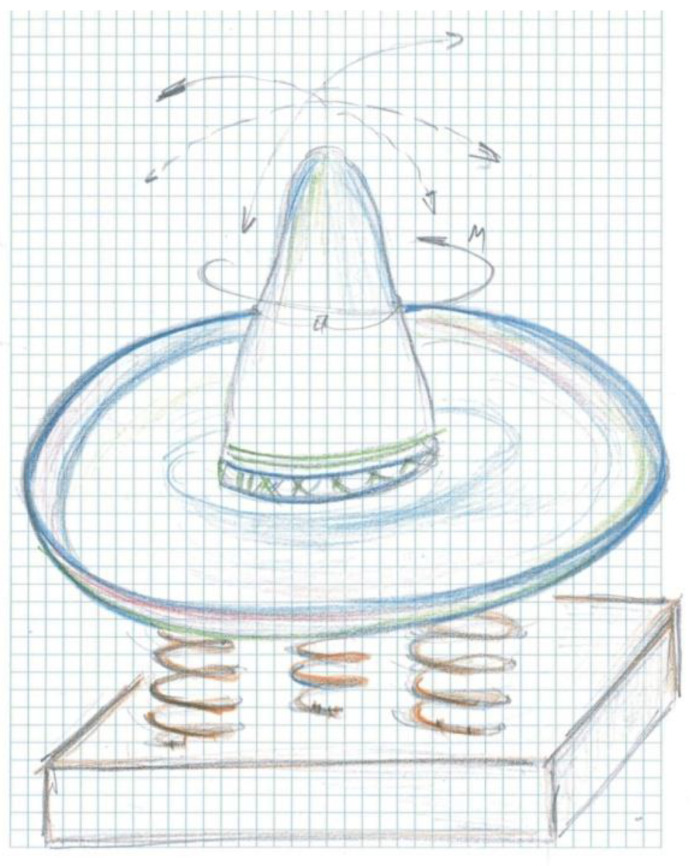
Concept sketch design of the sombrero’s hat.

Figure 16 presents the *sketched model* of the concept, identifying the particular elements presented above by the inspiration model, the sombrero hat.

*The model.* The extended surface of the border of the sun hat allows it to be requested for two to three children, the outer diameter is 120 cm, and on the surface of the cone, there are tie straps that children can use to establish a balance during the game.

The central axis is fixed at the base on support to which the three helical springs are fixed, and which provides limited pendulum movement produced kinematically by the children’s movement on the surface of the sombrero. The shaft is assembled by means of two rows of rolling bearings, a radial bearing at the base, and an axial bearing at the top.

*The material* proposed for the manufacture of the concept: are metal, fiberglass, and concrete.

## 8. Justification of The Choice of The Optimal Variant for The Carousel Design Concept

Taking into account the four variants proposed for the development of the concept, all technical and economic details are identified, correlated with the requirements imposed by the specifications and according to the score obtained, the final variant is chosen. This variant is to be identified with all the constructive details and further defined aspects related to materials and manufacturing technologies, as well as the identification of optimal solutions to develop a reliable product and harmoniously framed and integrated into the playgrounds or indoor recreation spaces.

In Table 2, the systemic, synoptic analysis applied to the four variants proposed for the carousel design concept is presented, including the following criteria associated with the variants.

Identifying the constructive characteristics imposed by the specifications, the justified variant chosen for the development of the concept is Variant 4, obtaining the highest score. Variant 4 is to be identified at a detail level so as to be able to identify all aspects related to functionality, manufacturing, and optimization from a technical–economic point of view.

**Figure 17:**
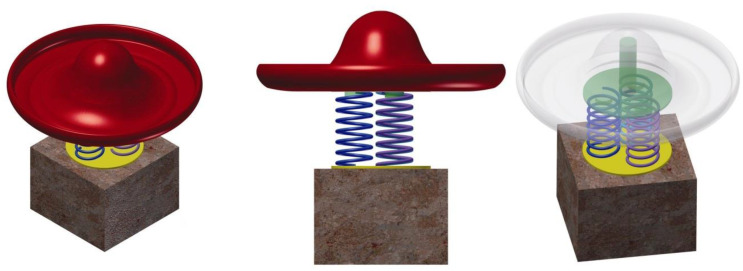
The outline principle, a 3D model of the optimal variant.

In Figure 17, the variant of the defined optimal solution can be identified as a draft, 3D model. The outline principle allows the primary identification of the structure, the gauge, and the proportion of the design concept, following the basis of this sketch, the concept of the carousel prototype will be finalized.

## 9. Design Concept, 3D Model

The design of the concept is carried out in the Autodesk Inventor environment, based on the sketch defined and justified from a technical and economic point of view, and the technical and technological solutions regarding the assembly of the design concept chosen as the final variant of the carousel will be identified.

The main element that underlies the concept is the sombrero sun hat. Obviously, from this aspect, the whole concept of the carousel will be defined. To begin with, the geometric shape of such a physical model is identified, starting from an orthogonal image imported into the AutoCAD environment. [2,3,13].

**Figure 18:**
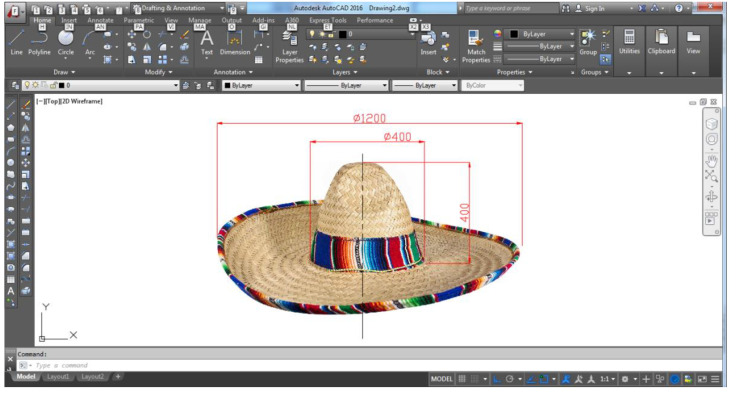
Scaling and defining the size dimensions of the sombrero hat model.

The geometric dimensions are defined, after which the AutoCAD environment is used to dimensionally correct the geometry of the sombrero structure. In Figure 18, the geometry and size of the sombrero sun hat are defined in the AutoCAD environment, identified by scaling the dimensions of the size, which subsequently defines the geometry of the hat profile in an axial section.

**Figure 19:**
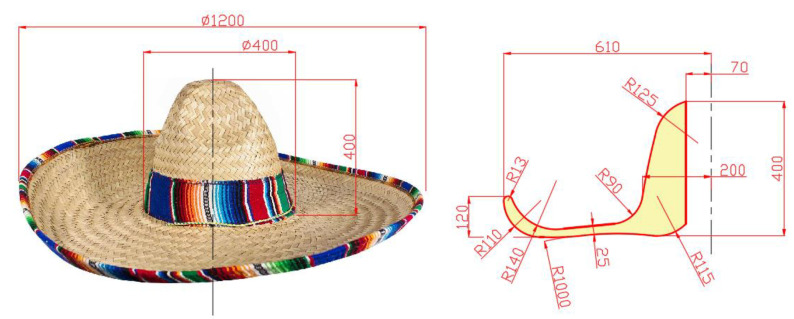
Definition of the geometry of the axial sector under investigation.

Thus, the design stages of the main landmark of the entire carousel assembly are structured, respectively, and the geometric shape of the sombrero will be finalized starting from a structure on frames linked together by a network of circular rings placed at equal distance on the perimeter direction of a base frame of the 12 frames that are radially arranged around the geometric axis of the sombrero sun hat. In the AutoCAD application, the geometry of such a frame is defined and it will be imported into the Autodesk Inventor application, where the 3D geometry of the structure is materialized. In Figure 19, the frame structure of the sombrero hat profile is shown, identifying the dimensioned geometry of the profile. [3,5,15].

In the Inventor application, the entire structure of rings arranged according to the perimeter geometry of the base frame is subsequently defined, defining the working planes associated with each ring. Technologically, the 12 frames will be made of 4 mm sheet metal defined by laser cutting technology, resulting in the geometric shape of Figure 19.

The 3D model is defined starting from a 2D sketch in which the profile defined in the AutoCAD application is imported, and the perimeter contour is closed, defined by a polyline, so it can be extruded. Since the two applications are compatible, it easily allows interaction between the work platform of the AutoCAD application, with the work application part of the Autodesk Inventor [2,6].

**Figure 20:**
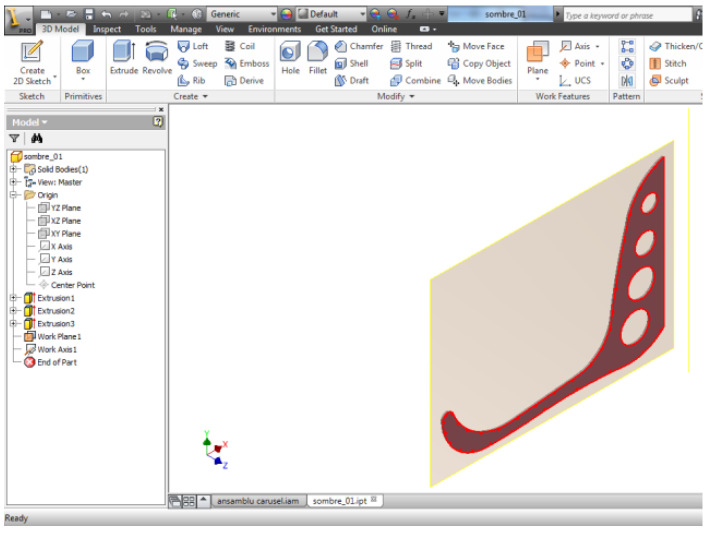
Part/Inventor environment.

**Figure 21:**
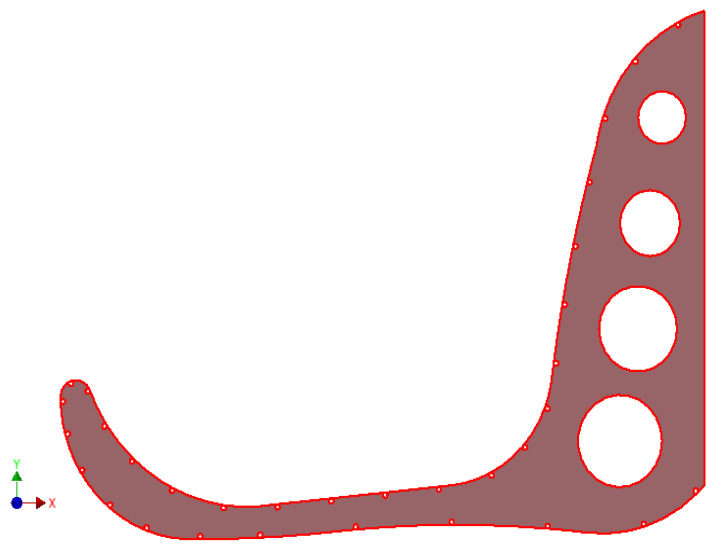
Details of the 3D model for axial sector.

Figure 20 and Figure 21 show the 3D model defined in the Autodesk Inventor Part application. Initially, a new work project is defined, so all components are associated with this new concept, and it is easily allowed to parameterize the benchmarks defined later in the Inventor application [44].

For further modeling of the structure, holes are highlighted perimetrically that serve to assemble with the ring structure, resulting in the final sombrero structure. On the front surface, holes are made for the relief of the defined geometry. You can see in Figure 21 the 3D modeling details.

**Figure 22:**
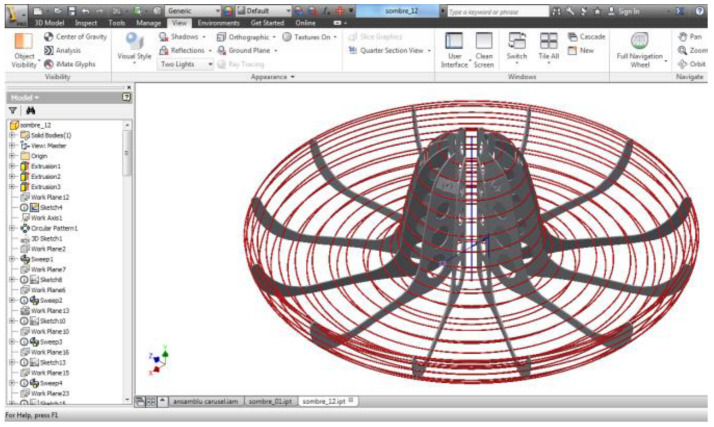
Three-dimensional model of sombrero ring structure.

The final 3D model of the sombrero sun hat is shown in Figure 22, the ring structure subsequently allows the “dressing” of the physical model with a structure of acrylic and synthetic fibers making up the final shape of the sombrero hat in layers and representing the external structure of the concept.

The covered structure of the model is defined using the Autodesk Inventor program but in the assembly design application. Thus, the structure modeled in the part application is imported into the Assembly application in order to later create the geometry of the outer layer that “dresses” the ring structure [2,5,9]. This application is used to facilitate the geometry import of the defining elements attached to the 3D modeled ring structure, initially in the part application.

For the physical model, a multilayer structure of acrylic material is recommended, combined with textile fabric (structure of glass fiber impregnated in the binder of acrylic nature), the thickness of the layer must exceed the thickness of the diameter of the ring wire, thus creating a block of increased strength on the entire generated surface. The coating operation is performed manually, there is no other technological process for the execution of this operation, which has a disadvantage related to production, such as a slight increase in cost due to manufacturing labor, which is usually high.

**Figure 23:**
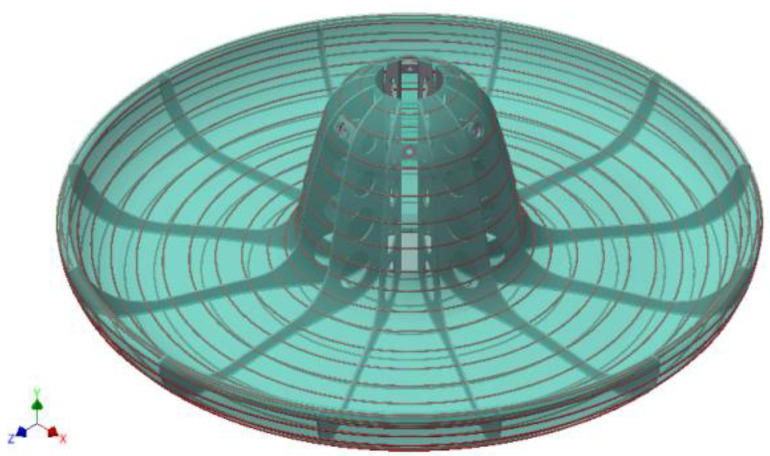
The concept of a sun hat, 3D model, Inventor.

In Figure 23, the resulting geometry is shown, the image shows the structure of concentric rings defined on the guiding curve, and the shell on the acrylic support wraps the carousel structure. The presentation mode has been chosen to be transparent in order to be able to observe the structure inside.

The design concept identifies several landmarks modeled and assembled in the Assembly/Inventor application, defined in turn, using constraint commands applied to defined work plans, as well as associated with different constraint options.

For the completion of the 3D Inventor model, we proceeded as much as possible to work in the assembly environment, in order to obtain at the end, the completely constrained design concept. The parameterized design was defined both in the Assembly Application and in the Part application of the Autodesk Inventor program, similarly, modeled as the working principle defined at the basic benchmark associated with the carousel.

**Figure 24:**
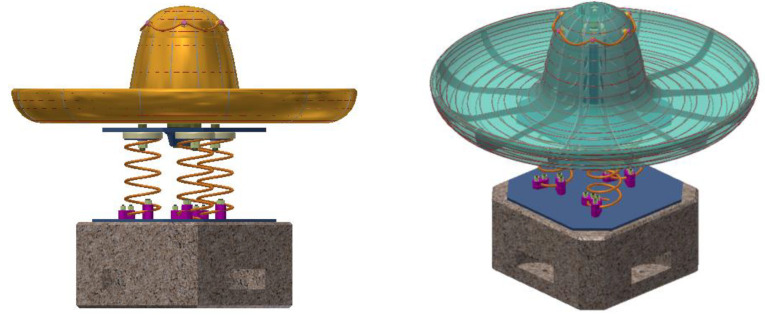
Design concept, roto-pendular carousel.

The conceptual design of the roto-pendular carousel Assembly is identified in Figure 24. Modeling of landmarks, as well as their assembly, were carried out in the Autodesk Inventor application. The design concept presents an optimized variant resulting from the systemic analysis applied to the design concept study and complies with the tasks imposed by the specification. Thus, the designed product identifies two main functions: rotational movement and pendulum movement, with respect to the proposed theme, respectively, the sombrero sun hat. For installation in playgrounds, there are no major installation difficulties, the concrete block is buried, access to the surface on the curved base of the product is easier, and in the case of indoor placement, the concrete block must be rigidly fixed by means of the studs attached to the base of the block on the four sides of the cube as you can see in Figure 24.

**Figure 25:**
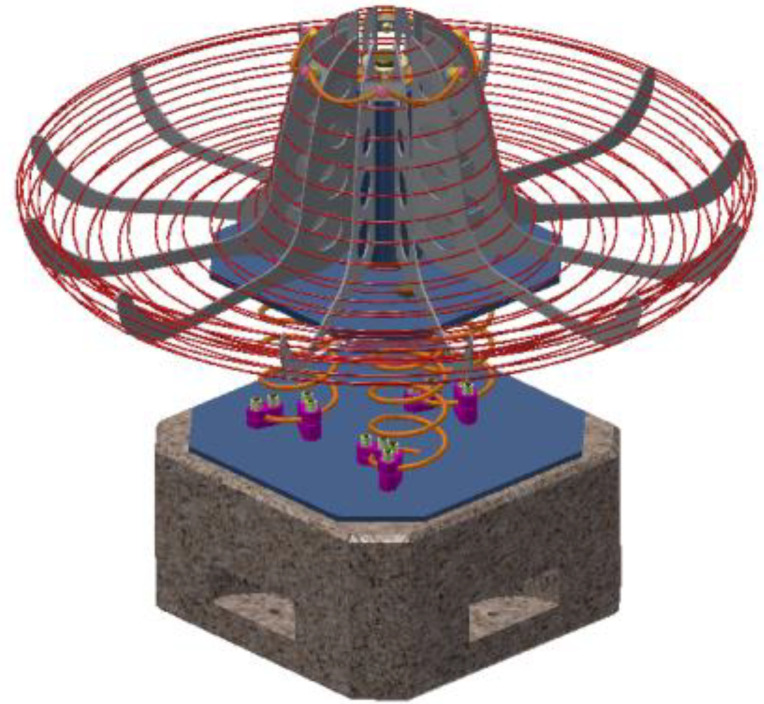
Prototype of the carousel concept, the wire mesh structure.

The structure of the sombrero hat is made of wire, identifying the sketched geometry, and the material to be covered is a multilayer structure of fiberglass that provides uniform coverage and gives additional resistance to the whole concept. The color is a combination of shades of yellow, red, green, and blue, to identify the originality of the sombrero. The wire mesh structure is fixed and non-removable on pipe support, and by means of rolling bearings, the entire structure is fixed on the base support This can be seen in Figure 25.

Thus, the carousel allows the definition of two functions imposed by the specification: rotational movement around the geometric axis and an undefined linear pendulum movement, due to the three springs symmetrically arranged at the base of the support and fixed in a rigid concrete structure at ground level.

**Figure 26:**
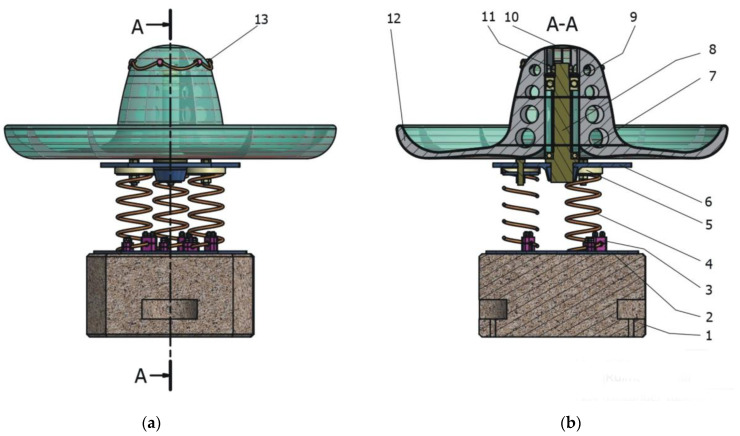
Prototype of carousel concept: (**a**) 1. concrete block structure, 2. lower support plate, 3. lower locking system, 4. helical spring, 5. upper fixing system, 6. upper fixed support plate, 7. roll-over bearing, 8. central axle-support, 9. axial bearing, 10. cap, 11. closing system, 12. subassembly structure of the sombrero hat, 13. safety system. (**b**) Section A-A.

**Figure 27:**
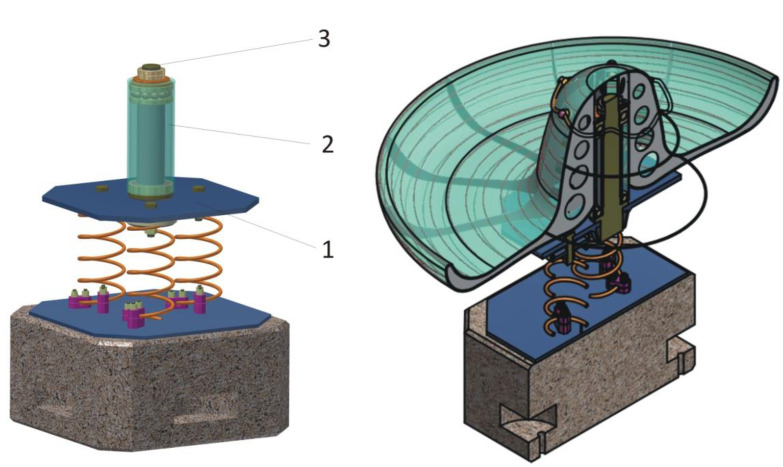
Detail of carousel concept (isometric view): 1. plate subassembly, 2. roll-over bearing assembly, 3. central axle support.

In Figure 26 and Figure 27, you can see the orthogonal axial section of the prototype concept.

**Figure 28:**
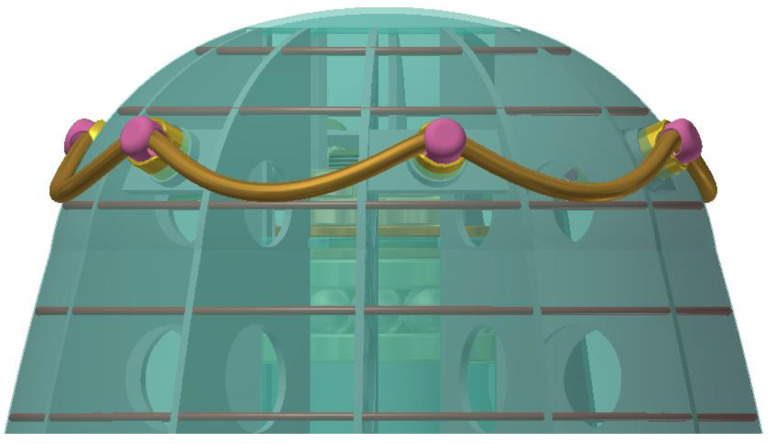
Detail of the safety rail attachment.

In Figure 28, you can see the detail of the subassembly safety rail support fixed up to the sombrero hat support of the final design concept of the carousel.

The fixing of the safety support is completed at the top in six points arranged radially, symmetrically, harmoniously, proportionally, and aesthetically completing the ergonomic shape of the entire prototype assembly.

## 10. Discussion and Conclusions

This article presented an integrated model of the innovation process that combines the conceptual variants of reference while defining an original solution applied to the considered case study. However, the work as a whole offers new scope for the management and deepening of the innovative conceptual design, as well as the exploration of engineering management in order to optimize the synthesis of innovative concepts [45].

The role and importance of the game lie in the fact that it facilitates the process of assimilation, fixation, and consolidation of knowledge, also due to its formative character that positively influences the development of the child’s personality. In this context, the materialization of the prototype concept proposed in this work also falls into place.

The final version of the prototype, following the systemic design analysis, proposes a new innovative approach to the carousel concept.

At the same time, the concept of the prototype associates two distinct functions, respectively: the function of rotation around its own axis and, at the same time, the pendulum function, within the limit of elasticity allowed by the helical springs that make the connection between the rotating platform and the block fixing base of stone.

Approaching the anatomical profile associated with the ergonomic shape of the sombrero hat structure, respectively, the sitting position allows the definition of an ergonomic anthropometric position in complete safety for the users.

The pendulum function is taken over automatically at the moment of rotation, especially when there is an imbalance of the moments or dynamic centrifugal forces, due to the weight variation of the children.

All things considered, the purpose of this article regarding the case study is not to provide academic evidence of the validity of the methodology, but rather to obtain practical experience and resources generating ideas, learning, and deepening of innovative technical solutions, applied to the analysis of products since from the design phase.

## Figures and Tables

**Table 1:** The main equipment that defines the prospectographic research study.

Nr.	Product	Functions / Features
1	Children’s spring swing [17] 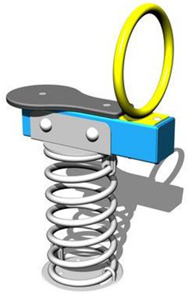	-Single seater, pendulum movement, undefined direction; -Colors in warm, complementary tones; -Robust, ensures a weight of up to 20 kg; -Intended for the age range of 3–7 years; -Rigid fastening in reinforced concrete block at the bottom, and at the top of the helical spring is fixed by means of two splints fixed on a metal plate, integral with the equipment seat; -Materials: 2”, 1” pipe, NDF seat support, moisture resistant -Adult supervision is recommended; -Lxwxh: dimensions: 800 × 300 × 1200
2	Bench Carousel [18] 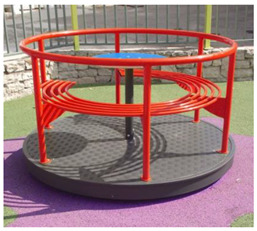	-Maximum capacity: 5 children; -Main functions: rotate, communicate; -The bench carousel is made of steel painted in an electrostatic field, having a high degree of reliability and resistance, aluminum platform. The carousel is equipped with electrostatic painted steel bench and HDPE central table; -Adult supervision is recommended; -Dimensions: 1550 × 1550 × 900; -Safe space: 6550 × 6550; -Installation surfaces: can be mounted on surfaces of: grass, slag, sand, concrete/asphalt, synthetic surfaces;
3	Fixed rotary Carousel [19] 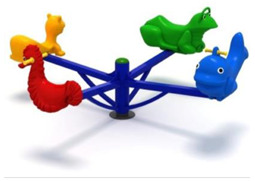	-The structure of the carousel is made of galvanized steel, polyethylene figurines; -Adult supervision is recommended; -Dimensions: 2200 × 2200 × 1000; -Safety space: 5200 × 5200 -Maximum capacity: 4 children; -Age category: 3 to 12 years; -This carousel can be mounted on surfaces of: grass, slag, sand, concrete/asphalt, synthetic surfaces.
4	Horse Carousel [20] 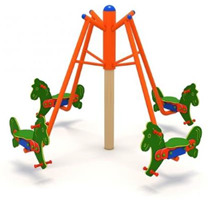	-This carousel has four seats. The equipment requires a generous seating area and is ideal for playgrounds within outdoor playgrounds, theme parks, adventure parks, outdoor/indoor playgrounds for children, adjacent to hotels and restaurants; - Dimensions: 2200 x 2200 x 1900; - age category: 3–12 years; -This carousel can be mounted on surfaces of: grass, slag, sand, concrete/asphalt, synthetic surfaces.
5	Classic Carousel [21] 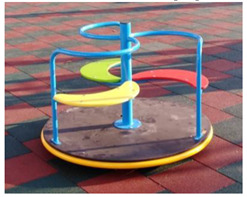	-The carousel is made of steel painted in electrostatic field, having a high degree of reliability and resistance, aluminum platform. -Maximum capacity: 3 children; -Age category: 3 to 12 years; -The carousel can be mounted on surfaces of: grass, slag, sand, concrete/asphalt, synthetic surfaces; -Dimensions: Ø 1250 mm × Height 930; -safe space: Ø 5250; -This carousel can be mounted on surfaces of: grass, slag, sand, concrete/asphalt, synthetic surfaces.
6	Firtree Carousel [22] 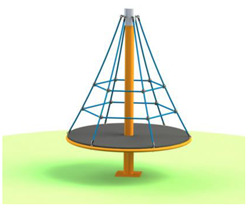	-The carousel is made of steel painted in electrostatic field, having a high degree of reliability and resistance, aluminum platform. -adult supervision is recommended; - Safe space: Ø 5500; -Age category: 3–12 years; -Three children -This carousel can be mounted on surfaces of: grass, slag, sand, concrete/asphalt, synthetic surfaces.
7	Carousel for people with disabilities [23] 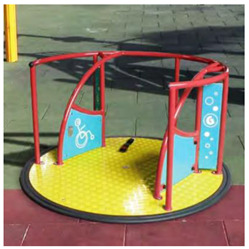	-This carousel for people with disabilities illustrates the safety system made of 2 adjustable straps for anchoring the wheelchair. The space required is of medium size, being perfect for outdoor playgrounds, outdoor playgrounds adjacent to nurseries and kindergartens, outdoor playgrounds for children adjacent to hotels and restaurants; -The carousel for people with disabilities is made of metal structure and HDPE panels; -Adult supervision is recommended; -Dimensions: 2200 × 2200 × 1900; -Age category: 3 to 12 years; -This carousel can be mounted on surfaces of: grass, slag, sand, concrete/asphalt, synthetic surfaces;
8	Fixed rotary Carousel [24] 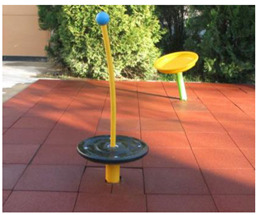	-The carousel is made of steel painted in electrostatic field, having a high degree of reliability and resistance, aluminum platform; -Maximum capacity: 3 children; -Age category: 3–12 years; -Dimensions: Ø 450mm × height 950; -Safe space: Ø 2450; -This carousel can be mounted on surfaces of: grass, slag, sand, concrete/asphalt, synthetic surfaces;
9	Air glider Carousel [25] 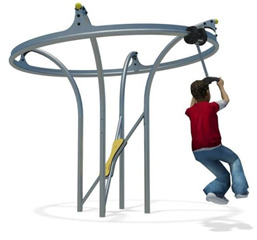	-The air glider playground equipment is a model that combines sports with games and is intended for playgrounds or theme parks. Children make physical efforts, agility and coordination are necessary in order to practice and experience the sensations generated by a zip line in combination with those of a carousel; -This element consists of a frame made of stainless steel, inclined handles and three flexible springs for swinging.The equipment requires a generous seating area and is ideal for play areas within: outdoor play areas, theme parks, adventure parks, outdoor playgrounds for children;
10	Roto-tilting carousel [26] 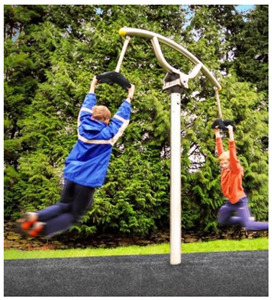	-This carousel model is a special design and is intended for playgrounds or theme parks. Children make physical efforts, and agility and coordination are necessary in order to practice and experience the sensations generated by a zip line in combination with those of a carousel. This element consists of a pole made of stainless steel, crossbar with full 360-degree rotation, and two handles. The equipment requires a generous location area and is ideal for play areas; -This carousel model has stainless steel structure.; -Age category: 10 to 16 years; -Size: 1850 × 300 × 2400; -Equipment weight: 84 kg; -Safe space: 8300 × 8300 × 3400; -Simultaneous users: 2 copies; -This carousel can be mounted on surfaces of: grass, slag, sand, concrete/asphalt, synthetic surfaces.
11	Carousel for children [27] 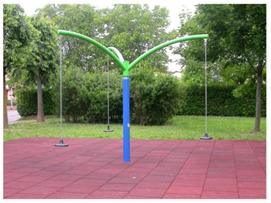	-This carousel model is a special design and is intended for playgrounds or theme parks. Children make physical efforts, and agility and coordination are necessary in order to practice and experience the sensations generated by a zip line in combination with those of a carousel.This carousel model is perfect for outdoor spaces, being very resistant, because in their manufacture stainless steel of a high quality is used, which will later give the child the degree of safety pursued; -This carousel model has stainless steel structure; -Adult supervision is recommended; -Dimensions: 3130 × 2740 × 2500; -Safe space: 9550 × 9550; -Simultaneous users: 2 children;
12	Spring swing Windsurfer [28] 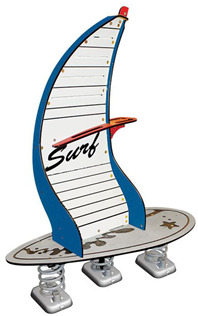	-Rigid fastening in reinforced concrete block at the bottom, and at the top of the three helical springs is fixed by means of two splints fixed on a metal plate, integral with the equipment plate; -Adult supervision is recommended; -Product dimensions: 1410 × 60 × 1870 mm; -Safe space: 4410 × 3600; -Simultaneous users: 2 children.
13	Seesaw [29] 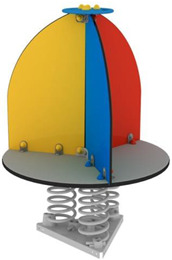	This seesaw requires for maximum three children to stand up on the plate, will have loads of fun if they cooperate; -Adult supervision is recommended; -Product dimensions: 920 × 920 × 1460 mm; -Simultaneous users: 3 children; -Age recomendation: 2 to 10 years; -Maximum fall height: 450 mm.

**Table 2:** Synoptic analysis applied to the four variants.

Variant	Functions	No. of Users	Materials	Safety	Options	Robust	Total
V1	1	4	1	80	1	25	112
V2	1	1	1	90	1	35	129
V3	2	2	2	75	1	20	102
V4	2	3	2	95	2	50	154

## Data Availability

Not applicable.
